# Leisure-Time Physical Activity, Sedentary Behaviour and Diet Quality are Associated with Metabolic Syndrome Severity: The PREDIMED-Plus Study

**DOI:** 10.3390/nu12041013

**Published:** 2020-04-07

**Authors:** Laura Gallardo-Alfaro, Maria del Mar Bibiloni, Catalina M. Mascaró, Sofía Montemayor, Miguel Ruiz-Canela, Jordi Salas-Salvadó, Dolores Corella, Montserrat Fitó, Dora Romaguera, Jesús Vioque, Ángel M. Alonso-Gómez, Julia Wärnberg, J. Alfredo Martínez, Lluís Serra-Majem, Ramon Estruch, José Carlos Fernández-García, José Lapetra, Xavier Pintó, Antonio García Ríos, Aurora Bueno-Cavanillas, José J. Gaforio, Pilar Matía-Martín, Lidia Daimiel, Rafael M. Micó-Pérez, Josep Vidal, Clotilde Vázquez, Emilio Ros, Cesar Ignacio Fernandez-Lázaro, Nerea Becerra-Tomás, Ignacio Manuel Gimenez-Alba, María Dolors Zomeño, Jadwiga Konieczna, Laura Compañ-Gabucio, Lucas Tojal-Sierra, Jéssica Pérez-López, M. Ángeles Zulet, Tamara Casañas-Quintana, Sara Castro-Barquero, Ana María Gómez-Pérez, José Manuel Santos-Lozano, Ana Galera, F. Javier Basterra-Gortari, Josep Basora, Carmen Saiz, Karla Alejandra Pérez-Vega, Aina M. Galmés-Panadés, Cristina Tercero-Maciá, Carolina Sorto-Sánchez, Carmen Sayón-Orea, Jesús García-Gavilán, Júlia Muñoz-Martínez, Josep A. Tur

**Affiliations:** 1 CIBER Fisiopatología de la Obesidad y Nutrición (CIBEROBN), Instituto de Salud Carlos III (ISCIII), 28029 Madrid, Spain; lauragala3@gmail.com (L.G.-A.); mar.bibiloni@uib.es (M.d.M.B.); catalinamaria95@hormail.es (C.M.M.); sofiamf16@gmail.com (S.M.); mcanela@unav.es (M.R.-C.); jordi.salas@urv.cat (J.S.-S.); dolores.corella@uv.es (D.C.); mfito@imim.es (M.F.); mariaadoracion.romaguera@ssib.es (D.R.); angelmago13@gmail.com (Á.M.A.-G.); jwarnberg@uma.es (J.W.); jalfredo.martinez@imdea.org (J.A.M.); lluis.serra@ulpgc.es (L.S.-M.); restruch@clinic.cat (R.E.); fjtinahones@hotmail.com (J.C.F.-G.); joselapetra543@gmail.com (J.L.); xpinto@bellvitgehospital.cat (X.P.); angarios2004@yahoo.es (A.G.R.); cvazquez@fjd.es (C.V.); EROS@clinic.cat (E.R.); cflazaro@unav.es (C.I.F.-L.); nerea.becerra@urv.cat (N.B.-T.); i.gimenez.alba@valencia.edu (I.M.G.-A.); mzomeno@imim.es (M.D.Z.); jadzia.koieczna@gmail.com (J.K.); lutojal@hotmail.com (L.T.-S.); jessicaperezlopez@uma.es (J.P.-L.); mazulet@unav.es (M.Á.Z.); tamara.nutricion@gmail.com (T.C.-Q.); scastro@clinic.cat (S.C.-B.); anamgp86@gmail.com (A.M.G.-P.); josemanuel.santos.lozano@gmail.com (J.M.S.-L.); agalera@idibell.cat (A.G.); javierbasterra@hotmail.com (F.J.B.-G.); jbasora.tgn.ics@gencat.cat (J.B.); carmen.saiz@uv.es (C.S.); kperez@imim.es (K.A.P.-V.); aina.galmes.panades@gmail.com (A.M.G.-P.); daisysorto2@yahoo.com (C.S.-S.); msayon@unav.es (C.S.-O.); jesusfrancisco.garcia@urv.cat (J.G.-G.); juliamm02@gmail.com (J.M.-M.); 2 Research Group on Community Nutrition & Oxidative Stress, University of Balearic Islands, 07122 Palma de Mallorca, Spain; 3 Health Research Institute of the Balearic Islands (IdISBa), 07120 Palma de Mallorca, Spain; 4 Department of Preventive Medicine and Public Health, IdISNA, University of Navarra, 31008 Pamplona, Spain; 5 Human Nutrition Unit, Biochemistry and Biotechnology Department, IISPV, Universitat Rovira i Virgili, 43201 Reus, Spain; 6 Department of Preventive Medicine, University of Valencia, 46100 Valencia, Spain; 7 Unit of Cardiovascular Risk and Nutrition, Institut Hospital del Mar de Investigaciones Médicas Municipal d’Investigació Mèdica (IMIM), 08003 Barcelona, Spain; 8 Nutritional Epidemiology Unit, Miguel Hernández University, Instituto de Investigación Sanitaria y Biomédica de Alicante (ISABIAL), 46020 Alicante, Spain; vioque@umh.es (J.V.); lcompan@umh.es (L.C.-G.); 9 CIBER Epidemiología y Salud Pública (CIBERESP), Instituto de Salud Carlos III (ISCIII), 28029 Madrid, Spain; abueno@ugr.es (A.B.-C.); mdelgado@ujaen.es (J.J.G.); 10 Bioaraba Health Research Institute, Osakidetza Basque Health Service, Araba University Hospital, University of the Basque Country UPV/EHU, 48013 Vitoria-Gasteiz, Spain; 11 Department of Nursing, School of Health Sciences, University of Málaga-IBIMA, 29071 Málaga, Spain; 12 Precision Nutrition Program, IMDEA Food, CEI UAM + CSIC, 28049 Madrid, Spain; 13 Department of Nutrition, Food Sciences, and Physiology, Center for Nutrition Research, University of Navarra, 31008 Pamplona, Spain; 14 Institute for Biomedical Research, University of Las Palmas de Gran Canaria, 35016 Las Palmas de Gran Canaria, Spain; 15 Department of Internal Medicine, IDIBAPS, Hospital Clinic, University of Barcelona, 08036 Barcelona, Spain; 16 Virgen de la Victoria Hospital, Department of Endocrinology, University of Málaga, 29010 Málaga, Spain; 17 Department of Family Medicine, Research Unit, Distrito Sanitario Atención Primaria Sevilla, 41013 Sevilla, Spain; 18 Lipids and Vascular Risk Unit, Internal Medicine, Hospital Universitario de Bellvitge, Hospitalet de Llobregat, 08907 Barcelona, Spain; 19 Lipids and Atherosclerosis Unit, Department of Internal Medicine, Maimonides Biomedical Research Institute of Cordoba (IMIBIC), Reina Sofia University Hospital, University of Cordoba, 14004 Cordoba, Spain; 20 Department of Preventive Medicine, University of Granada, 18071 Granada, Spain; 21 Department of Health Sciences, Centro de Estudios Avanzados en Olivar y Aceites de Oliva, University of Jaen, 23071 Jaen, Spain; 22 Department of Endocrinology and Nutrition, Instituto de Investigación Sanitaria Hospital Clínico San Carlos (IdISSC), 28040 Madrid, Spain; pilar.matia@gmail.com; 23 Nutritional Genomics and Epigenomics Group, IMDEA Food, CEI UAM + CSIC, 28049 Madrid, Spain; lidia.daimiel@imdea.org; 24 CIBER Diabetes y Enfermedades Metabólicas (CIBERDEM), Instituto de Salud Carlos III (ISCIII), 28029 Madrid, Spain; socochato68@gmail.com; 25 Network of Researchers REDI Fundación SEMERGEN, 28009 Madrid, Spain; 26 Department of Endocrinology, IDIBAPS, Hospital Clinic, University of Barcelona, 08036 Barcelona, Spain; jovidal@clinic.cat; 27 Department of Endocrinology, Fundación Jiménez-Díaz, 28040 Madrid, Spain; 28 Lipid Clinic, Department of Endocrinology and Nutrition, Institut d’Investigacions Biomèdiques August Pi Sunyer (IDIBAPS), Hospital Clínic, 08036 Barcelona, Spain; 29 Department of Medicine, Faculty of Medicine and Health Sciences, University of Barcelona, 08036 Barcelona, Spain; 30 Servicio Navarro de Salud, Osasunbidea. 31071 Pamplona, Spain; 31 Centro de Salud Raval, 03203 Elche, Spain; criterma@hotmail.es

**Keywords:** metabolic syndrome severity, physical activity, Mediterranean diet, depression risk, sedentary behaviour

## Abstract

Healthy lifestyle factors, such as physical activity (PA) and Mediterranean diet (MD), decrease the likelihood of developing metabolic syndrome (MetS). The aim of this study was to report main lifestyle components and related factors according to the MetS severity. Cross-sectional analysis was done of baseline lifestyle factors from 5739 participants with overweight/obesity and MetS features (aged 55–75 years) included in the PREDIMED-PLUS primary cardiovascular prevention randomized trial. Participants were categorized in tertiles according to a validated MetS severity score (MetSSS). Anthropometrics, visceral adiposity index, dietary nutrient intake, biochemical marker levels, as well as a Dietary Inflammatory Index and depression symptoms (Beck Depression Inventory-II) were measured. Diet quality was assessed using a 17-item energy-restricted MD questionnaire. Duration and intensity of PA was self-reported using the Minnesota-REGICOR Short Physical Activity Questionnaire. Sedentary behaviours were measured using the Spanish version of the Nurses’ Health Study questionnaire. The 30 s chair stand test was also assessed. Participants with highest MetSSS showed higher values of cardiovascular risk factors (except for total cholesterol and LDL cholesterol), depression risk, sedentary and TV viewing time, and lower moderate and vigorous leisure-time physical activity (LTPA). Highest MetSSS participants tended to a pro-inflammatory dietary pattern and tended to lower MD adherence. In addition, they showed lower carbohydrate and nut intake and higher intake of protein, saturated and trans fatty acids, cholesterol, iodine, sodium, red and processed meat products, other oils different from olive oil and spirit alcoholic drinks. The highest MetS severity score was associated with lower moderate and vigorous LTPA and higher sedentary time and depression risk, as they tended to a pro-inflammatory dietary pattern and lower MD adherence.

## 1. Introduction

Metabolic syndrome (MetS) is responsible for a 2.5-fold increased cardiovascular mortality, 5-fold increased risk of diabetes, 2-fold higher risk of coronary heart disease and cerebrovascular disease and 1.5-fold increase in the risk of all-cause mortality [1].

The prevalence of MetS is a major public health concern. Nearly 35% of all adults and 50% of those aged 60 years or older are estimated to have MetS in the US population [2] and nearly 31% in Spain [3]. This data is alarming, given the aging world population being a major contributor to the growing prevalence of MetS. Otherwise, the total cost of the MetS, including the cost of health care and loss of potential economic activity, is in trillions, and costs are likely to increase in the future.

The risk of developing MetS increases with the lack of physical activity (PA), which is also an important component of cardiovascular diseases (CVD) development [4,5]. Strong evidence shows that high amounts of sedentary behaviour have been associated with increased risk of several chronic conditions, mortality [6], and higher likelihood of MetS [7]. MetS has been bidirectionally associated with depression [8]. Diet has a potential role in the prevention and treatment of MetS; higher adherence to the Mediterranean diet (MD) can reduce cardiometabolic risk factors [9] and the prevalence of MetS, and cause reversion of this condition [10].

The binary nature of the MetS (presence/absence) does not allow for an exact estimation of the risk of MetS. In addition, certain components may be more strongly linked to MetS [11]. In this way, the MetS Severity Score (MetSSS) quantifies the cumulative amount of risk derived from the presence of MetS risk factors [12]. Given the lack of studies focused on the severity of the MetS in older populations, this study aimed to report leisure-time physical activity, sedentary behaviour, and diet quality according to the MetS severity.

## 2. Methods

### 2.1. Study Design

This research was a cross-sectional analysis of baseline data within the frame of the Prevención con Dieta Mediterránea (PREDIMED-Plus) trial, an ongoing six-year multicentre, parallel-group, randomised trial conducted in Spain, in order to evaluate the effect of a weight loss intervention programme based on an energy-restricted traditional MD, with physical activity promotion and behavioural support on cardiovascular disease morbimortality, compared with the usual care advice, consisting exclusively of an energy unrestricted traditional MD (control group). Details of the PREDIMED-Plus study protocol are fully described [13] and available at http://predimedplus.com/. The trial was registered in 2014 at the International Standard Randomized Controlled Trial (ISRCT; http://www.isrctn.com/ISRCTN89898870) with number 89898870.

### 2.2. Participants, Recruitment, Randomization, and Ethics

Eligible participants were community-dwelling adults (aged 55–75 in men; 60–75 in women; this sex–age range was chosen depending on the age that each gender is at high risk of suffering non-communicable diseases, the association of MetS with CVD, and the increasing prevalence of MetS with age), without documented history of CVD at enrolment, who were overweight or obese (body mass index (BMI) ≥ 27 and <40 kg/m^2^) and who met at least three criteria for MetS according to the updated harmonized definition of the International Diabetes Federation and the American Heart Association, and National Heart, Lung, and Blood Institute [14].

**Figure 1:**
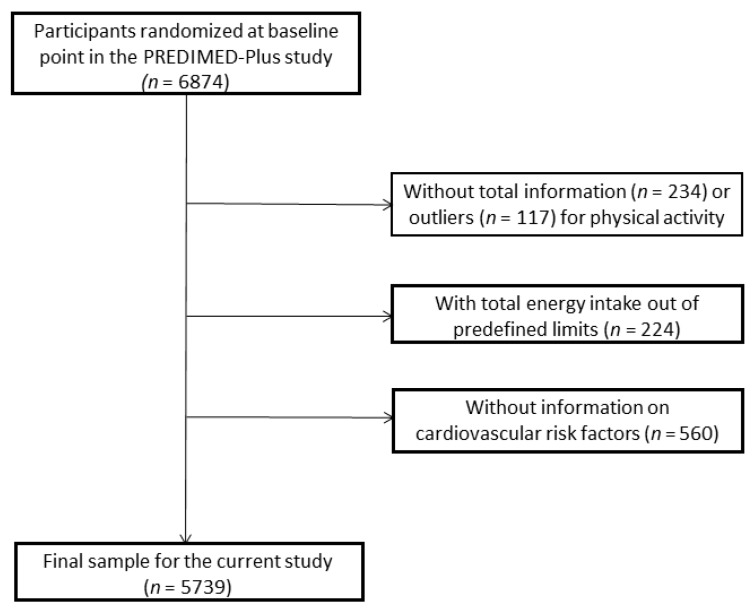
Flow-chart of the study participants.

From 5 September 2013 to 31 December 2016, a total of 9677 people was contacted, of which 6874 participants were recruited in 23 Spanish centres (universities, hospitals, and research institutes). However, the present analysis included 5739 subjects (2739 women) (Figure 1). Participants who did not respond to all physical activity (PA) questionnaires (*n* = 234) and participants reporting outliers for total PA (*n* = 117) expressed as metabolic equivalents of task (METs·min/week (at 3 or more standard deviations (SD) from the mean for each sex and age)) were excluded. Participants (*n* = 181) recording extreme total energy intakes (<500 or >3500 kcal/day in women or <800 or >4000 kcal/day in men), participants without information from food frequency questionnaires (FFQ) (*n* = 43), and those without total information on cardiovascular risk factors (*n* = 560) were also excluded.

All participants provided written informed consent. The study protocol and procedures were approved according to the ethical standards of the Declaration of Helsinki by all the participating institutions. The trial was registered at the International Standard Randomized Controlled Trial (ISRCTN: http://www.isrctn.com/ISRCTN89898870) with number 89898870 and registration date of 24 July 2014, retrospectively registered.

### 2.3. Anthropometric and Blood Pressure Measurements

Weight and height were measured by registered dietitians with calibrated scales and a wall-mounted stadiometer, respectively. BMI was calculated as weight in kilograms divided by the square of height in meters. Waist circumference (WC) was measured halfway between the last rib and the iliac crest by using an anthropometric tape. Systolic (SBP) and diastolic blood pressure (DBP) and heart rate (HR) were measured in triplicate with a validated semi-automatic oscillometer (Omron HEM-705CP, Lake Forest, IL, USA) after 5 min of rest in-between measurements while the participant was in a seated position. All anthropometric variables were determined in duplicate.

### 2.4. Blood Collection Analysis

Samples of fasting blood were collected after an overnight fast and biochemical analyses were performed on fasting plasma glucose, glycosylated haemoglobin (HbA1c), total cholesterol, high-density lipoprotein cholesterol (HDL-c), and triglycerides (TAG) concentrations in local laboratories using standard enzymatic methods, whereas low-density lipoprotein cholesterol (LDL-c) was calculated by the Friedewald formula.

### 2.5. Other Health Outcomes

Additional information related to sociodemographic and lifestyle aspects (education level, civil status, smoking habits, individual and family medical history, and current medication use) was collected. Furthermore, depressive symptoms were measured through the Beck Depression Inventory-II (BDI) validated in Spain. The Beck Depression Inventory-II includes 21 questions with four possible answers sorted according to depressive symptom severity, and scores range from 0 to 63 points [15].

### 2.6. Visceral Adiposity Index

Visceral adiposity was assessed by calculating the visceral adiposity index (VAI) according to previously validated equations [16]. VAI was defined as the following equations:

$$Men:\;\;VAI=\left(\frac{WC}{39.68+\left(1.88\times BMI\right)}\right)\times \left(\frac{TAG}{1.03}\right)\times \left(\frac{1.31}{HDL-c}\right)$$

$$Women:\;\;\;VAI=\left(\frac{WC}{36.58+\left(1.89\times BMI\right)}\right)\times \left(\frac{TAG}{0.81}\right)\times \left(\frac{1.52}{HDL-c}\right)$$

VAI was formulated assuming VAI = 1 for healthy non-obese subjects with normal adipose distribution and normal TAG and HDL-c levels.

### 2.7. Physical Activity and Sedentary Behaviour

Leisure time physical activity (LTPA) was measured using the Rapid Assessment of Physical Activity Questionnaires (RAPA-1 and RAPA-2) [17] and the validated Minnesota-REGICOR (Registre Gironí del Cor) Short Physical Activity questionnaire [18], including questions to collect information on the type of activity, frequency (number of days), and duration (min/day). METs were calculated by multiplying the intensity (showed by the MET score) and the duration spent on that activity (measured in minutes). The intensity was assigned based on the compendium of PA. According to PA intensity, activities were categorized into light PA (<4.0 MET), moderate PA (4–5.5 MET), and vigorous PA (≥6.0 MET).

The 30 s chair stand test was used as an indicator of lower-limb muscle strength. This test was done at the same desk that dietary assessment and anthropometric measurements were done, and was provided by registered technicians in physical activity. As reported, performance was based on the number of times participants could stand and sit on a chair in 30 s [19].

Sedentary behaviours were measured using the validated Spanish version of the Nurses’ Health Study questionnaire to assess sedentary behaviours [20], consisting of a set of open-ended questions assessing the average daily time spent over the last year in watching TV, sitting while using the computer, sitting on journeys (for work purposes or leisure time, as the driver or passenger in a car, subway, bus, employment status, retirement) and total sitting. Answers included 12 categories ranging from 0 to ≥9 h/day of sitting time for the corresponding activity. Furthermore, participants reported their average daily sleeping time for both weekdays and weekends, using the non-validated open question, “How many hours do you sleep on average per day on weekdays and weekends?”

### 2.8. Dietary Assessment

Registered dietitians collected data on dietary intake with a semi-quantitative 143-item FFQ, assessing dietary habits over the previous 12 months, validated in Spain [21]. Detailed information about the development, reproducibility, and validity of FFQ in the PREDIMED cohort has been previously reported. For each item, a typical portion size was included, and consumption frequencies were registered in nine categories that ranged from “never or almost never” to “≥6 times/day”. Energy and nutrient intakes were calculated as frequency multiplied by nutrient composition of the specified portion size for each food item, using a computer program based on available information in food composition tables [22]. The selected frequency item was converted to a daily intake. For example, if a response was 5–6 times a week, it was converted to 0.78 servings per day (5.5 times/week). For each FFQ food item, we estimated the average amount of food consumed (grams), the average total energy intake, and the average intake of a set of macro- and micro-nutrients by computing the mean of the values for the individual foods assigned to that item. We also considered the total nutrient intake, and the average intake of micronutrients from dietary supplements declared by participants in the FFQ.

### 2.9. Assessment of Mediterranean Diet Adherence

Participants were also administered a 17-item Mediterranean dietary questionnaire, a modified version of the previously validated questionnaire used in the PREDIMED trial designed to assess adherence to MD. Compliance with each of the 17 items relating to characteristic food habits was scored with 1 and 0 points. Therefore, the total score range was 0–17, with 0 meaning no adherence and 17 meaning maximum adherence [23].

### 2.10. Dietary Inflammatory Index

The Dietary Inflammatory Index (DII) is a score used to assess the inflammatory potential of a diet, as previously described [24]. The score reports the effect of 45 food parameters on six inflammatory biomarkers (interleukins (IL-1B, IL-4, IL-6, IL-10), Tumour Necrosis Factor-alpha (TNF-α), and highly sensitive C-Reactive Protein [CRP]), consisting of whole foods, nutrients, and other bioactive compounds derived from a much larger literature review. The food parameters obtained a positive score according to whether its effect was pro-inflammatory (significantly increased IL-1β, IL-6, TNF-α, or CRP, or decreased IL-4 or IL-10), a negative score if its effect was anti-inflammatory, and 0 if no significant change in inflammatory biomarkers was found. The DII is based on dietary intake derived from the validated FFQ used in the PREDIMED-Plus trial. The individual intake of each food parameter was subtracted from a global standard database, and then divided by the world standard deviation for each food parameter. These values were converted to a percentile score, each percentile was doubled, and then 1 was subtracted to achieve a symmetrical distribution (from −1 to +1 and centred on 0). Afterwards, each one of these values was multiplied by the overall food parameter specific inflammatory score. Finally, the sum of all the food parameter specific DII scores provided the overall DII score for everyone. Thus, positive DII scores represent a pro-inflammatory diet and negative DII scores represent an anti-inflammatory diet. In this way, we considered the following food parameters to calculate the DII: alcohol, carbohydrate, cholesterol, energy, iron, fibre, folic acid, garlic, green/black tea, magnesium, monounsaturated fatty acids (MUFA), omega-3 fatty acids (w-3 FA), omega-6 fatty acids, niacin, onion, protein, polyunsaturated fatty acids (PUFA), riboflavin, saturated fatty acids (SFA), selenium, thiamine, total fat, trans fatty acids (TFA), vitamin A, vitamin B12, vitamin B6, vitamin C, vitamin D, vitamin E, and zinc.

### 2.11. Metabolic Syndrome Severity Score

The Metabolic Syndrome Severity Score (MetSSS) was calculated as previously described [12]. A MetSSS value of zero indicates that subjects were at or below clinical thresholds for all MetS risk factors (WC, TAG, HDL, SBP, DBP, glucose). Similarity in scores across sex, age, and medication subgroups, confirms the validity of using this standard, robust, and generalizable formula [12]. MetSSS was categorized in tertiles, being the cut-off points 2.7 and 4.0. The first tertile (T1) represents the sample with the lowest severity of risk factors for cardio-metabolic disease and the third tertile (T3) represents the sample with the highest severity of risk factors for cardio-metabolic disease.

### 2.12. Statistics

Analyses were performed with the SPSS statistical software package version 25.0 (SPSS Inc., Chicago, IL, USA). Qualitative data are shown as prevalence, and differences across tertiles of MetSSS were examined using the chi-square test. Quantitative data are shown as mean, standard deviation (SD), or median and interquartile range (IQR). In Table 1, normality of data was assessed using the Kolmogorov–Smirnov test and, as age was non-normally distributed, the Kruskal–Wallis test was used to compare differences in means among tertiles using Bonferroni post-hoc correction. In Table 2, Table 3, Table 4 and Table 5, differences in means among tertiles of MetSSS were tested by one-factor analysis of variance (ANOVA). Equality of variances was assessed with Levene’s test. In addition, analysis of covariance (ANCOVA) was used in order to assess the difference in means among tertiles of MetSSS, adjusted for potential confounding. Bonferroni post-hoc correction was used for multiple comparisons to control type I error. Results were considered statistically significant if the *p*-value (2 tailed) <0.05.

## 3. Results

Table 1 shows general characteristics of participants according to tertiles of MetSSS. There were significant differences in all variables, except for hypertriglyceridemia. Specifically, the percentage of women and the prevalence of obesity, high blood pressure (BP), hyperglycaemia, low HDL cholesterol and abdominal obesity, and the prescription of antihypertensive agents, anti-cholesterol agents, insulin, oral hypoglycaemic agents, and aspirin or antiplatelet drugs increased with increasing MetS severity, whereas the education level decreased. Women showed significantly higher MetSSS compared to men (3.68 ± 1.37 vs. 3.25 ± 1.44, *p* < 0.001). Participants with lowest MetS severity included a higher percentage of those who were married, workers, and former smokers, whereas participants with the highest MetSSS included a lower percentage of married and retired participants, current smokers, and a higher percentage of non-workers; 58.0% of non-smokers were women, representing 93.5% of women in T3, vs. 38.8% of non-smokers women in T1, representing 91.3% of total women in T1.

Cardiovascular risk factors and BDI according to tertiles of MetSSS are shown in Table 2. After full adjustment, there were significant differences in all variables among tertiles, except for total cholesterol and LDL-c. Weight, BMI, WC, glucose, HbA1C, TAG, SBP, DBP, HR, and VAI increased significantly with increasing MetS severity. BDI was higher in T3 (tertile with the highest MetSSS), and HDL-c was higher in T1. 

PA parameters and sedentary behaviour are shown in Table 3. The 30 s chair stand test decreased with increasing MetS severity. Total sedentary and TV-viewing time were significantly lower in T1 (tertile with the lowest MetSSS) compared with T3, whereas total, moderate, and vigorous LTPA were significantly higher. No significant differences were found in light LTPA and sleeping time. 

Table 4 shows nutrient intake characteristics of the total sample according to tertiles of MetSSS. After full adjustment, SFAs were significantly lower in T1 participants, whereas carbohydrate intake was significantly higher. T3 participants showed higher intake of protein, total fat, SFA, TFA, cholesterol, iodine, and sodium compared with T1. After adjusting for sex and age, w-3 FA intake was significantly lower in T3 compared to T1, but this significance was lost when adjusting for all demographic variables. No significant differences were found in total energy, MUFA, PUFA, linoleic acid, glycaemic index, fibre, alcohol, vitamin and mineral intake, except for iodine and sodium.

Table 5 shows food consumption, DII, and adherence to MD of participants according to tertiles of MetSSS. After full adjustment, total meat intake significantly decreased with decreasing MetS severity. In addition, red and processed meat intake was significantly lower in T1 compared to T3, whereas nut intake was significantly higher. T3 participants showed significantly lower intake of nuts and significantly higher intake of red and processed meat, and oils different from olive oil and spirit alcoholic drinks, compared with T1. After adjusting for sex and age, MD adherence was statistically significantly higher in T1 compared to T3 participants, whereas DII and white meat intake were significantly lower in T1, compared with the positive and significantly higher DII in T3 participants, but this significance was lost when adjusting for all demographic variables. No differences were found in fruits, vegetables, potatoes, legumes, cereals, fish and seafood, eggs, dairy products, olive oil, snacks, and wine and beer intake.

## 4. Discussion

To the best of our knowledge, this is the first study that has examined LTPA, sedentary behaviour, and dietary characteristics in older adults with MetS according to the MetS severity score (MetSSS). The most relevant observation of this study was that participants with the highest MetSSS (T3) showed higher levels of sedentary time and depression risk and lower total LTPA, and they also tended to have a pro-inflammatory dietary pattern and tended to have lower MD adherence. Conversely, participants with the lowest MetSSS (T1) showed lower levels of sedentary time and depression risk and higher total LTPA, and they tended to have an anti-inflammatory dietary pattern and tended to have higher adherence to MD. Concretely, T3 participants showed lower carbohydrate and nut intake and moderate and vigorous LTPA, which is associated with higher risk of CVD [4,5,25]. Conversely, T3 showed higher intake of protein, SFA, TFA, cholesterol, as well as higher consumption of red and processed meat, and other oils different from olive oil and spirits compared with T1, resulting in an increased risk of several major chronic diseases [26,27].

### 4.1. Sociodemographic Factors

Previous evidence has shown an association between married individuals and a lower incidence of CVD and coronary heart disease (CHD) and lower CHD and stroke mortality compared with unmarried individuals [28], which is in agreement with our results, where a majority of the participants with lower levels of MetSSS were married. Our results in which women showed higher levels of MetSSS is in agreement with previous observations in which women showed a higher risk of CV mortality attributed to the higher prevalence of MetS in elderly women [29] and the distribution of central adiposity, insulin resistance, and hormonal regulation [30]. In accordance with our results showing that retired participants were less frequent in the highest MetSSS tertiles, previous observations have associated retirement with increasing LTPA [31]. Smoking is associated with MetS and its individual components, and smoking cessation is beneficial to MetS [32]. This is contrary to our results in which those participants with the highest MetSSS were mainly non-smokers, perhaps due to a higher percentage of women in T3 participants. Previous evidence has shown that women smoke less than men [33], which is in accordance with our results. Educational level decreased with increasing MetSSS, which is in accordance with previous observations [34] in which an inverse association between educational level and MetS was found.

### 4.2. Cardiovascular Risk Factors

An increase in cardiovascular risk factors (weight, BMI, WC, glucose, HbA1c, TAG, SBP, and DBP) with increasing MetSSS and an increase in HDL-c in participants with lower MetSSS were similar to previous findings, in which the MetSSS improved prediction of diabetes and CHD [11]. Furthermore, resting HR is an indicator of the autonomic nervous system. High HR represents an imbalance in the central nervous system, which is closely associated with insulin resistance. Resting HR increased as MetSSS did, and this agrees with previous observations that have suggested an increased risk of MetS with increasing HR [35]. In addition, VAI is a novel mathematical model to estimate visceral adiposity and is a good indicator of adipose tissue dysfunction, being the optimal VAI cut-off value around 1.0. High VAI values are associated with poor future metabolic outcomes and is related with higher cardiometabolic risk [16]. This correlates with our results in which VAI increased with increasing MetSSS. Despite that, total cholesterol and LDL cholesterol tended to be lower in participants with higher MetSSS, though after adjusting for the medication used, this significance disappeared.

### 4.3. Depressive Symptoms

Depressive symptoms are associated with prevalent and incident frailty in the older population, which is associated with activities of daily living impairment, hospitalization, and death. Our analysis on depressive symptoms showed that risks of depression increased with increasing MetS severity, which is in accordance with previous results that show a bidirectional association between depression and MetS [8]. Other authors suggested that psychological characteristics, especially depression, may increase risks of MetS [36]. Comparing our results with previous findings [37], our participants showed a higher risk of depression, especially among MetSSS participants of highest severity.

### 4.4. Sedentary Behaviour and Physical Activity

Strong evidence has shown that high amounts of sedentary behaviour are associated with increased risks of several chronic conditions and mortality [5,6,38]. Prolonged TV viewing time is associated with an increased risk of type 2 diabetes mellitus (T2DM), CVD, and all-cause mortality [36]. Other authors showed that prolonged sedentary time is independently associated with deleterious health outcomes, regardless of PA [6]. Such results are strongly related to our findings, in which sedentary time and TV-viewing were higher in the highest MetSSS participants. In addition, our results are similar to previous observations in which total sedentary time increased as the number of MetS components did [39], and greater levels of sedentary time, independent of other levels of PA, was associated with being metabolically unhealthy and high levels of sedentary time were strongly related to a higher likelihood of MetS [7]. 

Previous evidence has shown that PA decreases the risk of developing MetS and is an important component of CVD prevention [40]. Conversely, the lack of PA is a major cause of chronic diseases and suggests an inverse relationship between PA level and MetS, obesity, non-alcoholic fatty liver disease, and T2DM. An excess of weight and lack of PA are two important determinants of MetS [4]. Lack of PA explains a greater amount of the variance of MetS than any other factors of lifestyle, education, sex, and family history, and substantially mitigates the strong association of age and BMI with MetS [12,41]. Previous findings have also shown that high levels of moderate-intensity PA seem to decrease the increased risk of death associated with high sitting time, and to mitigate the increased risk associated with high levels of TV-viewing time [42]. Therefore, all these findings agree with our results (lack of PA among the highest MetSSS subjects).

Lower-body strength is important for the maintaining of functional mobility and preventing or delaying the onset of disability, and the chair stand test provides information on declines in mobility in older adults and is a measure used to identify frail individuals [19]. Compared to non-institutionalized Spanish older adults [43], our highest MetSSS participants showed less repetitions in the chair stand test, and thus, less low-body strength. 

### 4.5. Dietary Characteristics

MD is characterized by high consumption of fruits and vegetables, legumes, nuts and whole cereals, and a high intake of olive oil, but low-to-moderate consumption of dairy products, low intake of meat and poultry, and regular, but moderate intake of wine [44]. MD prevents the development of CVD, breast cancer, depression, colorectal cancer, diabetes, obesity, asthma, and cognitive decline [42], and decreases prevalence of MetS and cardiometabolic risk factors [9] but can also reverse MetS conditions [10]. Such results are related to our findings which showed a tendency towards higher MD adherence in participants with the lowest MetSSS. The evidence shows that food comprising MD has a cardioprotective effect, improving cardiovascular health and reducing CVD morbidity and mortality. This effect is caused by improving vascular function (improving hyperemia indices, reducing inflammation, improving platelet function, and enhancing nitric oxide (NO) utilization and availability) and lipid profiles (reducing LDL-c, TG, increasing HDL-c, and reducing LDL-oxidation), reducing BP (improving vascular function, reducing inflammation, reducing reactive oxygen species (ROS), and enhancing NO utilization), reducing oxidative stress (reducing LDL-oxidation, improving anti-oxidant capacity, reducing ROS, and reducing isoprostanes) and reducing weight (reducing ROS, lipid profile and BP, and improving exercise capacity) [44]. This is in accordance with the tendency of highest MetSSS participants towards a lower MD adherence.

Inflammation is an underlying pathophysiological process in chronic diseases, such as obesity, T2DM, and CVD. A pro-inflammatory diet is associated with increased all-cause mortality. In addition, pro-inflammatory diets are associated with an increased risk of MetS [45]. Our results also agree these previous findings, as well as that a pro-inflammatory state is one component of MetS [46].

Our results also agree previous findings in which total, red, and processed meat consumption is positively associated with MetS [27], which could potentially increase the risk of chronic diseases, including T2DM, CVD, and several types of cancer [47]. Conversely, our lowest severity MetSSS participants tended to have a higher intake of white meat, like previous findings that showed an inverse association between white meat intake and MetS [28]. 

The higher intakes of total protein, total fat, SFA, TFA, and cholesterol showed by the highest MetSSS may have been due to the high consumption of total, red, and processed meat and other oils and fats different from olive oil, and it is consistent with previous results in which the quality and the amount of FA intake were associated with risk of MetS [48]. Previous findings showed a decrease in cardiovascular risk on reductions of SFA intake [49], like our results on lower SFA intake in lower severity MetSSS participants. Previous evidence has shown a positive association between TFA intake and the development of CVD [50], which is in accordance with our results. Omega-3 FA decreases the production of inflammatory mediators, having a positive effect on obesity and T2DM, decreasing the appearance of CVD risk factors, and conferring cardioprotection due to their blood pressure lowering and anti-inflammatory properties [51]. Our findings on highest MetSSS participants that tended to have lower ω-3 FA intake may be understood under this umbrella.

Carbohydrate intake stimulates insulin secretion, promoting fat storage and strongly inhibiting adipose tissue lipolysis and fatty acid oxidation. In this way, low-carbohydrate diets seem to ameliorate insulin resistance and MetS [52]. Our results showed, however, that higher levels of carbohydrate intake in participants led to the lowest levels of MetSSS risk, but it is important to highlight that the kind of carbohydrate is of considerable importance, where fruits, vegetables, legumes, and whole grains are appropriate sources of carbohydrates with cardioprotective components. Our results on lower intakes of nuts among the highest MetSSS subjects are in accordance with previous findings that pointed out how a higher consumption of nuts was inversely associated with total CVD and CHD [25], and nut consumption, especially walnuts, showed a significant, inverse association with MetS risk [53]. Despite dairy products being one of the richest sources of iodine [54], their intake is not significantly increased in highest MetSSS subjects. Moreover, consumption of other iodine sources [fish, fruits, and vegetables] were no different between participants with different MetSSS. Accordingly, the higher iodine and sodium intake among the highest MetSSS participants could be related with the increased consumption of processed meat products [55] in these participants, also associated with higher CVD risk [56]. 

Finally, the relationship between alcohol consumption and CVD is complex and controversial. High alcohol consumption is associated with both dyslipidaemia and high glycosylated haemoglobin, [57] and alcohol abuse has been shown to increase mortality and cardiovascular risk [26]. These observations are in accordance with our findings in which participants with the highest MetSSS showed a higher intake of spirits.

### 4.6. Strengths and Limitations

The main strength of this study was that, to our knowledge, it is the first study to examine lifestyle factors, such as LTPA, dietary characteristics, sedentary behaviour, and depressive symptoms in older adults with MetS according to MetS severity. Another strength of our study is that a large sample of older adults living in the Mediterranean area with MetS was assessed. In addition, the FFQ used to collect information on nutrient intake took nutritional supplements, such as multivitamins and minerals, into consideration as a contribution to micronutrient intake. However, supplement use could also be considered as a confounding factor. The study also has several limitations. First, although the FFQ is a validated tool, it could overestimate the intake of certain food groups. Second, the use of self-reported data to evaluate PA has inherent limitations, as questionnaires overestimate the engagement in PA. Third, this cross-sectional study limits the ability to elucidate a causal relationship between MetS and lifestyle factors. 

## 5. Conclusions

Older adults with most severe MetS showed lower moderate and vigorous LTPA and higher levels of sedentary time and depression risk and tended to have a pro-inflammatory dietary pattern and lower MD adherence. As this was an observational study, future studies will need to elucidate the causal relationship between the severity of MetS and lifestyle factors. This approach provided new and useful information for determining prevention and treatment plans in a population as heterogeneous as patients with MetS, according to the severity of the disease.

## Figures and Tables

**Table 1:** Baseline characteristics of participants according to tertiles of the Metabolic Syndrome Severity Score (MetSSS).

Variables	Tertile 1 (*n* = 1913)	Tertile 2 (*n* = 1913)	Tertile 3 (*n* = 1913)	*p*-Value
Women, *n* (%)	704 (36.8)	981 (51.3)	1054 (55.1)	<0.001
Age, years	64.7 ± 5.1 ^a^	65.3 ± 4.9 ^a^	65.0 ± 4.9	<0.001
Prevalence of obesity, *n* (%)	1000 (52.3)	1535 (80.2)	1647 (86.1)	<0.001
Education level, *n* (%)	1899	1893	1895	<0.001
Illiterate or primary education	846 (44.5)	945 (49.9)	992 (52.3)	
Secondary education	573 (30.2)	552 (29.2)	524 (27.7)	
Academic or graduate	480 (25.3)	396 (20.9)	379 (20.0)	
Smoking habit, *n* (%)	1904	1905	1909	<0.001
Never smoked	749 (39.3)	852 (44.7)	904 (47.4)	
Former smoker	907 (47.6)	791 (41.5)	795 (41.6)	
Current smoker	248 (13.0)	262 (13.8)	210 (11.0)	
Married status	1902	1906	1910	0.012
Single or divorced	242 (12.7)	261 (13.7)	261 (13.7)	
Married	1499 (78.8)	1442 (75.7)	1427 (74.7)	
Widower	161 (8.5)	203 (10.7)	222 (11.6)	
Employment status, *n* (%)	1902	1904	1895	<0.001
Working	455 (23.9)	342 (18.0)	367 (19.4)	
Non-working	372 (19.6)	435 (22.8)	508 (26.8)	
Retired	1075 (56.5)	1127 (59.2)	1020 (53.8)	
MetS components, *n* (%)				
High blood pressure Hyperglycaemia Hypertriglyceridemia Low HDL-cholesterol Abdominal obesity	1719 (89.9) 1322 (69.1) 1065 (55.7) 742 (38.8) 1769 (92.5)	1750 (91.5) 1435 (75.0) 1072 (56.0) 837 (43.8) 1859 (97.2)	1801 (94.1) 1558 (81.4) 1102 (57.6) 903 (47.2) 1876 (98.1)	<0.001 <0.001 0.440 <0.001 <0.001
Medication				
Antihypertensive agents	1460 (76.3)	1466 (76.6)	1539 (80.4)	0.003
Anti-cholesterol agents	966 (50.5)	964 (50.4)	1053 (55.0)	0.018
Insulin	36 (1.9)	64 (3.3)	174 (9.1)	<0.001
Oral hypoglycaemic agents	271 (14.2)	433 (22.6)	802 (41.9)	<0.001
Aspirin or antiplatelet drugs	240 (12.5)	278 (14.5)	376 (19.7)	<0.001

Data is presented as mean ± SD or as number of participants (percentage). Abbreviations: BMI: body-mass-index; HDL: high density lipoprotein. Differences in percentages were tested by chi-squared test and difference in means between tertiles were tested by Kruskal Wallis test for non-normally distributed variables with Bonferroni post-hoc correction. Differences (*p*-value <0.05) between ^a^ Tertile 1 vs. Tertile 2.

**Table 2:** Cardiovascular risk factors and Beck Depression Inventory-II according to tertiles of Metabolic Syndrome Severity Score (MetSSS).

Variables	Tertile 1 (*n* = 1913)		Tertile 2 (*n* = 1913)		Tertile 3 (*n* = 1913)		*p*-Value	Sex and Age Adjusted *p*-Value	Full Adjusted *p*-Value
	Mean ± SD	Median (IQR)	Mean ± SD	Median (IQR)	Mean ± SD	Median (IQR)			
Weight, kg	81.6 ± 11.0 ^a,b^	81.2 (16.1)	86.5 ± 11.9 ^a,c^	85.3 (16.9)	91.6 ± 13.9 ^b,c^	90.6 (19.0)	<0.001	<0.001	<0.001
BMI, kg/m^2^	30.4 ± 2.3 ^a,b^	30.1 (3.3)	32.7 ± 3.0 ^a,c^	32.5 (4.3)	34.4 ± 3.6 ^b,c^	34.6 (5.7)	<0.001	<0.001	<0.001
Waist circumference, cm	101.7 ± 7.2 ^a,b^	102.0 (10.7)	107.7 ± 7.7 ^a,c^	107.0 (10.6)	113.1 ± 10.0 ^b,c^	113.0 (13.3)	<0.001	<0.001	<0.001
Women	94.8 ± 4.3 ^a,b^	95.0 (6.5)	103.4 ± 5.0 ^a,c^	104.0 (6.4)	110.4 ± 9.0 ^b,c^	112.0 (11.4)	<0.001	<0.001	<0.001
Men	105.7 ± 5.3 ^a,b^	106.0 (7.4)	112.3 ± 7.4 ^a,c^	113.5 (11.0)	116.4 ± 10.1 ^b,c^	116.0 (16.1)	<0.001	<0.001	<0.001
Glucose, mg/dL	101.6 ± 12.5 ^a,b^	101.0 (17.0)	109.3 ± 17.8 ^a,c^	106.0 (24.0)	129.4 ± 40.4 ^b,c^	118.0 (48.0)	<0.001	<0.001	<0.001
Glycated haemoglobin,%	5.8 ± 0.5 ^a,b^	5.7 (0.5)	6.0 ± 0.6 ^a,c^	5.9 (0.7)	6.5 ± 1.2 ^b,c^	6.2 (1.4)	<0.001	<0.001	<0.001
Total cholesterol, mg/dL	196.0 ± 35.8	195.0 (50.0)	197.8 ± 38.2	196.0 (52.0)	194.3 ± 38.4	192.0 (51.0)	<0.001	<0.001	<0.001
HDL-cholesterol, mg/dL	49.2 ± 12.0 ^a,b^	47.0 (15.0)	47.8 ± 11.4 ^a^	46.0 (14.0)	46.8 ± 12.1 ^b^	46.0 (15.0)	<0.001	<0.001	<0.001
LDL-cholesterol, mg/dL	121.8 ± 41.2	119.0 (43.0)	122.8 ± 46.3	120.0 (44.0)	117.4 ± 40.5	114.0 (44.0)	<0.001	<0.001	<0.001
Triglycerides, mg/dL	133.4 ± 49.5 ^a,b^	127.0 (68.0)	151.0 ± 64.1 ^a,c^	138.0 (77.0)	171.8 ± 103.3 ^b,c^	144.0 (94.0)	<0.001	<0.001	<0.001
Systolic blood pressure, mmHg	134.1 ± 14.2 ^a,b^	133.3 (18.3)	138.5 ± 16.3 ^a,c^	137.3 (20.7)	143.9 ± 18.8 ^b,c^	142.0 (23.7)	<0.001	<0.001	<0.001
Diastolic blood pressure, mmHg	80.1 ± 8.4 ^a,b^	80.3 (11.0)	81.7 ± 9.5 ^a,c^	81.7 (12.7)	84.1 ± 10.8 ^b,c^	83.3 (15.0)	<0.001	<0.001	<0.001
Heart rate, bpm	68.0 ± 9.9 ^a,b^	67.3 (13.0)	70.3 ± 10.5 ^a,c^	69.7 (14.0)	73.0 ± 11.2 ^b,c^	72.0 (14.9)	<0.001	<0.001	<0.001
Visceral adiposity index	2.0 ± 0.9 ^a,b^	1.8 (1.3)	2.5 ± 1.4 ^a,c^	2.2 (1.6)	3.0 ± 2.5 ^b,c^	2.4 (2.1)	<0.001	<0.001	<0.001
Beck Depression Inventory-II	7.5 ± 6.8 ^b^	6.0 (9.0)	8.4 ± 7.4 ^c^	7.0 (9.0)	9.5 ± 7.9 ^b,c^	8.0 (9.0)	<0.001	<0.001	<0.001

Abbreviations: BMI: body mass index; CI: confidence interval. Differences among tertiles of MetS Severity Score were tested by 1-factor ANOVA (*p*-value) and ANCOVA, adjusted *p*-value for sex and age, and full adjusted p-value for sex, age, education level, smoking habit, married and employment status and medication used. Bonferroni post-hoc correction was used for multiple comparisons. Differences (full adjusted *p*-value <0.05) between ^a^ Tertile 1 vs. Tertile 2, ^b^ Tertile 1 vs. Tertile 3, ^c^ Tertile 2 vs. Tertile 3.

**Table 3:** Physical activity parameters and sedentary behaviour according to tertiles of MetS Severity Score.

Varibles	Tertile 1 (*n* = 1913)		Tertile 2 (*n* = 1913)		Tertile 3 (*n* = 1913)		*p*-Value	Sex and Age Adjusted *p*-Value	Full Adjusted *p*-Value
	Mean ± SD	Median (IQR)	Mean ± SD	Median (IQR)	Mean ± SD	Median (IQR)			
Sedentary time, h/d	7.9 ± 1.9 ^a,b^	8.0 (2.7)	8.0 ± 1.9 ^a^	8.0 (2.9)	8.2 ± 2.0 ^b^	8.0 (3.3)	<0.001	<0.001	<0.001
TV-viewing time, h/d	5.0 ± 1.7 ^b^	5.0 (2.0)	5.2 ± 1.8	5.0 (2.3)	5.3 ± 1.9 ^b^	5.0 (2.4)	<0.001	<0.001	0.003
Sleeping time, h/d	7.0 ± 1.2	7.0 (2.0)	7.1 ± 1.2	7.0 (2.0)	7.0 ± 1.3	7.0 (2.0)	0.128	0.056	0.081
Total LTPA, MET·min/d	378.4 ± 282.3 ^a,b^	311.7 (360.8)	335.0 ± 272.4 ^a^	267.2 (353.3)	307.3 ± 261.1 ^b^	239.8 (303.7)	<0.001	<0.001	<0.001
Light LTPA, MET·min/d	115.3 ± 140.9	71.9 (159.8)	110.3 ± 132.6	63.9 (159.8)	110.7 ± 126.3	79.9 (151.9)	0.433	0.588	0.417
Moderate LTPA, MET·min/d	147.7 ± 195.7 ^b^	74.9 (224.8)	128.0 ± 183.9	40.0 (199.8)	111.0 ± 181.9 ^b^	10.0 (159.8)	<0.001	<0.001	<0.001
Vigorous LTPA, MET·min/d	115.4 ± 184.8 ^a,b^	18.0 (163.3)	96.7 ± 164.8 ^a^	12.0 (137.2)	85.7 ± 155.8 ^b^	8.0 (105.6)	<0.001	<0.001	<0.001
30-s chair stand test, *n*	14.1 ± 5.0 ^a,b^	14.0 (6.0)	13.2 ± 5.0 ^a,c^	13.0 (5.0)	12.5 ± 4.9 ^b,c^	12.0 (5.0)	<0.001	<0.001	<0.001

Abbreviations: IQR: interquartile range; LTPA: leisure time physical activity; s: second; SD: standard deviation. Differences among tertiles of MetS Severity Score were tested by 1-factor ANOVA (*p*-value) and ANCOVA, adjusted p-value for sex and age, and full adjusted *p*-value for sex, age, education level, smoking habit, married and employment status. Bonferroni post-hoc correction was used for multiple comparisons. Differences (full adjusted *p*-value <0.05) between ^a^ Tertile 1 vs. Tertile 2, ^b^ Tertile 1 vs. Tertile 3, ^c^ Tertile 2 vs. Tertile 3.

**Table 4:** Energy and nutrient intake according to tertiles of Metabolic Syndrome Severity Score (MetSSS).

Vriables	Tertile 1 (*n* = 1913)		Tertile 2 (*n* = 1913)		Tertile 3 (*n* = 1913)		*p*-Value	Sex and Age Adjusted *p*-Value	Full Adjusted *p*-Value
	Mean ± SD	Median (IQR)	Mean ± SD	Median (IQR)	Mean ± SD	Median (IQR)			
Total energy (TE), kcal/d	2392.7 ± 547.8	2354.1 (724.4)	2359.0 ± 553.9	2329.5(758.7)	2345.7 ± 549.1	2321.2 (764.6)	0.024	0.733	0.640
Total fat intake, %TE	39.0 ± 6.3 ^a,b^	39.0 (8.7)	39.8 ± 6.6 ^a^	39.8 (8.7)	39.6 ± 6.5 ^b^	39.6 (9.1)	0.001	0.002	0.003
MUFA, % TE	20.2 ± 4.6 ^a^	20.1 (6.0)	20.7 ± 4.7 ^a^	20.6 (6.4)	20.4 ± 4.6	20.2 (6.5)	0.005	0.012	0.023
PUFA, %TE	6.4 ± 1.8	6.1 (2.2)	6.4 ± 1.8	6.1 (2.3)	6.3 ± 1.9	6.0 (2.2)	0.450	0.415	0.457
SFA,%TE	9.8 ± 2.0 ^a,b^	9.6 (2.6)	10.0 ± 2.0 ^a^	9.9 (2.5)	10.1 ± 2.0 ^b^	10.0 (2.6)	0.001	<0.001	<0.001
Trans FA, g/d	0.6 ± 0.4 ^b^	0.5 (0.5)	0.6 ± 0.4	0.5 (0.5)	0.6 ± 0.4 ^b^	0.5 (0.5)	0.225	0.056	0.029
Linoleic acid, g/d	13.8 ± 5.6	12.8 (7.0)	13.6 ± 5.5	12.7 (7.3)	13.4 ± 5.7	12.3 (6.8)	0.194	0.805	0.830
w-3 FA, g/d	2.4 ± 0.9	2.3 (1.3)	2.4 ± 1.0	2.2 (1.3)	2.3 ± 0.9	2.1 (1.2)	0.005	0.014	0.049
Carbohydrate intake, %TE	41.1 ± 6.7 ^a,b^	41.1 (9.4)	40.4 ± 6.9 ^a^	40.4 (9.2)	40.5 ± 6.9 ^b^	40.5 (9.4)	0.008	<0.001	<0.001
Glycaemic index	54.1 ± 5.1	54.4 (6.8)	53.7 ± 5.2	54.1 (6.8)	53.9 ± 5.1	54.2 (6.7)	0.146	0.367	0.557
Protein intake, %TE	16.5 ± 2.8 ^b^	16.2 (3.6)	16.8 ± 2.8	16.7 (3.7)	17.1 ± 2.9 ^b^	16.9 (3.6)	<0.001	0.002	0.003
Cholesterol, mg/d	382.1 ± 118.8 ^b^	369.5 (136.9)	380.3 ± 119.3	168.8 (143.3)	386.4 ± 113.4 ^b^	374.5 (135.9)	0.251	0.013	0.006
Fibre intake, g/d	26.3 ± 9.0	24.7 (11.0)	26.3 ± 9.0	25.0 (11.0)	25.9 ± 8.4	25.0 (10.6)	0.300	0.061	0.094
Alcohol intake, g/d	12.3 ± 15.3	6.2 (15.4)	10.4 ± 14.1	4.7 (13.0)	10.4 ± 15.6	4.3 (11.9)	<0.001	0.509	0.457
Vitamin A, µg/d	1111.9 ± 639.7	936.8 (781.5)	1098.8 ± 654.4	929.0 (753.1)	1120.0 ± 638.8	936.7 (766.4)	0.592	0.583	0.480
Vitamin B1, mg/d	1.6 ± 0.4	1.6 (0.5)	1.6 ± 0.4	1.6 (0.6)	1.6 ± 0.4	1.6 (0.5)	0.935	0.840	0.742
Vitamin B2, mg/d	2.0 ± 0.6	1.9 (0.8)	2.0 ± 0.7	1.9 (0.8)	2.0 ± 0.6	1.9 (0.8)	0.271	0.619	0.497
Vitamin B3, mg/d	40.6 ± 10.0	40.0 (13.8)	40.8 ± 10.0	40.4 (12.9)	41.0 ± 9.9	40.5(12.7)	0.386	0.219	0.151
Vitamin B6, mg/d	2.4 ± 0.6	2.3 (0.8)	2.4 ± 0.6	2.3 (0.8)	2.3 ± 0.6	2.3 (0.8)	0.283	0.237	0.344
Vitamin B9, µg/d	352.5 ± 104.8	336.8 (134.3)	353.5 ± 103.7	338.1 (126.7)	349.3 ± 99.4	339.0 (125.8)	0.419	0.222	0.279
Vitamin B12, µg/d	10.0 ± 4.6	9.2 (5.8)	10.0 ± 4.4	9.1(5.5)	10.1 ± 4.5	9.2(5.6)	0.400	0.200	0.212
Vitamin C, mg/d	200.2 ± 87.8	184.5(108.1)	204.7 ± 86.3	188.6(114.7)	200.2 ± 83.7	187.0 (104.6)	0.166	0.138	0.156
Vitamin D, µg/d	6.3 ± 3.5	5.2 (5.4)	6.2 ± 3.4	5.2 (5.4)	6.1 ± 3.5	5.0 (5.0)	0.230	0.189	0.232
Vitamin E, mg/d	10.6 ± 4.0	10.0 (4.3)	10.6 ± 3.9	10.0 (4.3)	10.6 ± 4.1	9.9 (4.3)	0.966	0.733	0.646
Calcium, mg/d	1026.7 ± 339.7	984.8 (431.9)	1040.9 ± 348.3	997.3 (453.8)	1038.1 ± 350.2	997.4 (449.6)	0.409	0.475	0.320
Phosphorus, mg/d	1749.5 ± 411.4	1715.6 (540.9)	1765.1 ± 431.6	1733.7 (597.4)	1769.1 ± 422.0	1736.1 (558.2)	0.315	0.294	0.149
Magnesium, mg/d	423.1 ± 109.0	409.5 (142.6)	422.6 ± 111.2	410.9 (143.9)	417.1 ± 106.3	405.5 (145.7)	0.171	0.241	0.372
Iron, mg/d	16.6 ± 4.0	16.3 (5.4)	16.5 ± 4.0	16.2 (5.1)	16.4 ± 3.9	16.1 (5.2)	0.355	0.958	0.999
Iodine, µg/d	274.1 ± 154.8 ^b^	256.5 (130.5)	288.3 ± 162.7	258.9 (140.4)	290.8 ± 158.8 ^b^	259.7 (125.6)	0.002	0.030	0.019
Potassium, mg/d	4457.1 ± 1067.4	4346.5 (1401.0)	4493.8 ± 1101.2	4396.4 (1441.6)	4466.5 ± 1077.9	4365.2 (1374.5)	0.552	0.686	0.700
Selenium, µg/d	117.6 ± 33.4	114.9 (42.7)	116.6 ± 33.4	114.2 (44.5)	117.6 ± 32.4	115.4 (43.1)	0.545	0.428	0.280
Zinc, mg/d	13.2 ± 3.3	12.9 (4.3)	13.2 ± 3.3	12.9 (4.5)	13.2 ± 3.2	13.0 (4.2)	0.969	0.400	0.257
Sodium, mg/d	2427.8 ± 760.3 ^b^	2332.5 (957.0)	2406.6 ± 794.1 ^c^	2312.1 (1008.0)	2459.4 ± 775.8 ^b,c^	2371.3 (976.7)	0.106	0.001	<0.001

Abbreviations: IQR: interquartile range; MUFA: monounsaturated fatty acid; PUFA: polyunsaturated fatty acid; SD: standard deviation; SFA: saturated fatty acid; w-3 FA: omega-3 fatty acid. Differences among tertiles of MetS Severity Score were tested by 1-factor ANOVA (p-value) and ANCOVA, adjusted *p-value* for sex and age and full adjusted p-value for sex, age, education level, smoking habit, married and employment status. Bonferroni post-hoc correction was used for multiple comparisons. Differences (full adjusted p-value < 0.05) between ^a^ Tertile 1 vs. Tertile 2, ^b^ Tertile 1 vs. Tertile 3, ^c^ Tertile 2 vs. Tertile 3.

**Table 5:** Food consumption, Dietary Inflammatory Index, and adherence to Mediterranean Diet according to tertiles of Metabolic Syndrome Severity Score (MetSSS).

Variables	Tertile 1 (*n* = 1913)		Tertile 2 (*n* = 1913)		Tertile 3 (*n* = 1913)		*p*-Value	Sex and Age Adjusted *p*-Value	Full Adjusted *p*-Value
	Mean ± SD	Median (IQR)	Mean ± SD	Median (IQR)	Mean ± SD	Median (IQR)			
**Food groups**									
Fruits, g/d	352.3 ± 203.4	320.4 (245.1)	361.6 ± 204.9	331.8 (246.2)	358.0 ± 210.0	325.6 (252.0)	0.373	0.880	0.863
Vegetables, g/d	324.5 ± 138.1	304.3 (181.9)	329.6 ± 143.2	306.5 (170.8)	328.7 ± 139.7	306.9 (177.8)	0.487	0.825	0.758
Potatoes and tubers, g/d	64.9 ± 42.5	50.0 (67.1)	65.7 ± 43.0	50.0 (67.1)	65.2 ± 43.5	50.0 (67.1)	0.858	0.789	0.746
Legumes, g/d	21.0 ± 11.0	20.0 (9.7)	20.8 ± 11.7	20.6 (13.1)	20.6 ± 11.1	17.1 (12.6)	0.670	0.205	0.811
Nuts, g/d	15.9 ± 17.9 ^b^	10.3 (21.7)	15.3 ± 17.6 ^c^	8.6 (23.6)	13.5 ± 16.5 ^b,c^	8.0 (17.1)	<0.001	<0.001	<0.001
Cereals, g/d	153.4 ± 79.3	126.4 (112.5)	147.8 ± 77.3	119.7 (116.0)	150.3 ± 74.5	126.4 (112.5)	0.080	0.411	0.441
Whole cereals	41.9 ± 64.1	5.0 (75.0)	42.3 ± 63.6	5.0 (75.0)	41.1 ± 62.4	4.0 (75.0)	0.831	0.173	0.307
Refined cereals	111.5 ± 89.1	92.1 (162.1)	105.5 ± 85.5	87.6 (161.2)	109.3 ± 85.5	92.1 (160.7)	0.097	0.110	0.185
Fish and seafood, g/d	101.9 ± 47.0	97.0 (64.0)	103.5 ± 49.3	99.5 (65.3)	102.0 ± 47.5	96.0 (62.1)	0.504	0.557	0.757
White fish	38.9 ± 25.6	25.4 (42.9)	40.3 ± 27.0	25.4 (42.9)	40.4 ± 26.4	25.4 (42.9)	0.160	0.690	0.570
Bluefish	35.0 ± 22.8	25.7 (37.1)	34.7 ± 22.5	25.7 (38.2)	34.0 ± 22.7	25.7 (34.1)	0.354	0.253	0.311
Seafood	28.0 ± 21.2	30.6 (17.3)	28.6 ± 23.8	30.6 (17.3)	27.6 ± 21.3	30.6 (17.3)	0.425	0.321	0.376
Total meat, g/d	144.7 ± 59.8 ^b,a^	138.5 (71.1)	147.2 ± 57.4 ^c,a^	143.0 (70.8)	152.0 ± 59.0 ^b,c^	146.9 (74.7)	<0.001	<0.001	<0.001
Red meat	48.5 ± 35.1 ^a,b^	41.4 (46.2)	49.5 ± 34.0^a^	41.4 (46.2)	49.4 ± 34.2 ^b^	42.8 (50.0)	0.596	0.004	0.002
White meat	60.3 ± 33.5	64.3 (42.9)	61.6 ± 33.8	64.3 (42.9)	64.2 ± 35.7	64.3 (52.9)	0.002	0.029	0.066
Processed meat	33.8 ± 22.0 ^b^	30.6 (23.0)	34.1 ± 22.5 ^c^	31.3 (24.2)	36.2 ± 24.9 ^b,c^	32.4 (25.1)	0.002	<0.001	<0.001
Viscera	2.2 ± 5.3	0.0 (0.0)	2.1 ± 5.4	0.0 (0.0)	2.3 ± 5.3	0.0 (0.0)	0.517	0.295	0.328
Eggs	24.0 ± 12.4	25.7 (0.0)	23.6 ± 12.0	25.7 (0.0)	24.2 ± 11.5	25.7 (0.0)	0.300	0.190	0.155
Dairy products, g/d	332.5 ± 194.4	298.2 (189.4)	349.3 ± 203.3	310.2 (265.0)	351.6 ± 206.4	304.6 (297.9)	0.006	0.125	0.077
Whole-fat dairy	45.5 ± 97.1	0.0 (53.6)	43.8 ± 96.5	0.0 (53.6)	45.3 ± 97.9	0.0 (53.6)	0.829	0.522	0.541
Skimmed dairy	146.9 ± 160.5	125.0 (200.0)	156.8 ± 170.8	125.0 (209.5)	155.4 ± 175.1	111.5 (209.5)	0.147	0.572	0.471
Cheese	29.8 ± 25.1	24.8 (32.4)	30.5 ± 24.6	24.8 (28.8)	30.9 ± 25.0	25.0 (29.1)	0.387	0.470	0.287
Dairy desserts	16.0 ± 30.2	6.7 (15.3)	14.4 ± 25.5	6.7 (15.3)	15.2 ± 32.5	6.7 (15.3)	0.272	0.574	0.534
Cookies and sweets, g/d	26.9 ± 29.5	18.9 (32.7)	25.6 ± 28.6 ^c^	15.5 (30.7)	27.5 ± 30.7 ^c^	17.8 (33.7)	0.108	0.081	0.041
Olive oil, g/day	39.2 ± 17.1	50.0 (25.0)	40.2 ± 16.8	50.0 (25.0)	39.0 ± 17.1	50.0 (25.0)	0.048	0.030	0.054
Other oils and fats, g/d	2.8 ± 6.2 ^b^	0.7 (2.2)	2.8 ± 6.2 ^c^	0.7 (3.0)	3.4 ± 7.0 ^b,c^	0.8 (4.3)	0.004	0.003	0.007
Ready-to-eat-meals/Snacks, g/d	10.9 ± 14.1	5.3 (16.0)	10.7 ± 14.8	4.3 (16.7)	11.0 ± 14.6	5.3 (16.0)	0.790	0.306	0.195
Alcoholic drinks, ml/d									
Wine	67.9 ± 107.5	14.3 (100.0)	54.3 ± 96.1	14.3 (78.6)	53.7 ± 100.7	6.7 (49.5)	<0.001	0.413	0.437
Beer	119.9 ± 216.8	22.0 (141.4)	112.0 ± 224.5	22.0 (141.4)	102.8 ± 222.4	22.0 (141.4)	0.056	0.226	0.294
Spirits	3.7 ± 10.8 ^b^	0.0 (3.3)	3.0 ± 9.5 ^c^	0.0 (3.3)	3.9 ± 12.6 ^b,c^	0.0 (3.3)	0.020	0.001	0.001
**Dietary Inflammatory Index**	−0.1 ± 2.0	−0.1 (3.1)	0.0 ± 2.1	0.0 (3.0)	0.1 ± 2.0	0.1 (3.1)	0.103	0.035	0.057
**Mediterranean Diet Adherence**	8.5 ± 2.7	8.0 (4.0)	8.5 ± 2.7	8.0 (3.0)	8.4 ± 2.5	8.0 (3.0)	0.345	0.037	0.068

Abbreviations: IQR: interquartile range; SD: standard deviation. Differences among tertiles of MetS Severity Score were tested by 1-factor ANOVA (*p*-value) and ANCOVA, adjusted *p*-value for sex and age, and full adjusted p-value for sex, age, education level, smoking habit, married and employment status. Bonferroni post-hoc correction was used for multiple comparisons. Differences (full adjusted *p*-value <0.05) between ^a^ Tertile 1 vs. Tertile 2, ^b^ Tertile 1 vs. Tertile 3, ^c^ Tertile 2 vs. Tertile 3.

## Data Availability

There are restrictions on the availability of data for the PREDIMED-Plus trial, due to the signed consent agreements around data sharing, which only allow access to external researchers for studies following the project purposes. Requestors wishing to access the PREDIMED-Plus trial data used in this study can make a request to the PREDIMED-Plus trial Steering Committee chair: jordi.salas@urv.cat. The request will then be passed to members of the PREDIMED-Plus Steering Committee for deliberation.

## Abbreviations

ANCOVA	analysis of covariance
ANOVA	analysis of variance;
BMI	body mass index
BP	blood pressure;
CHD	coronary heart disease
CVD	cardiovascular disease
DBP	diastolic blood pressure
DII	dietary inflammatory index
FA	fatty acid
FFQ	food frequency questionnaire
HDL-c	high-density lipoprotein cholesterol
HR	heart rate
IQR	interquartile range
LDL-c	low-density lipoprotein cholesterol
MD	Mediterranean diet
MetS	Metabolic Syndrome
MetSSS	metabolic syndrome severity score
MUFA	monounsaturated fatty acids
NO	nitric oxide
PREDIMED	PREvención con DIeta MEDiterránea
PA	physical activity
PUFA	polyunsaturated fatty acids
RAPA	Rapid Assessment of Physical Activity Questionnaires
ROS	reactive oxygen species
SBP	systolic blood pressure
SD	standard deviations
SFA	saturated fatty acids
TAG	triglycerides
TFA	trans fatty acid
T2DM	type 2 diabetes mellitus
VAI	visceral adiposity index
WC	waist circumference
ω-3 FA	omega-3 fatty acids

## References

[1] Engin A. (2017). The definition and prevalence of obesity and metabolic syndrome. Advances in Experimental Medicine and Biology.

[2] Aguilar M., Bhuket T., Torres S., Liu B., Wong R.J. (2015). Prevalence of the Metabolic Syndrome in the United States, 2003–2012. JAMA.

[3] Fernández-Bergés D., Cabrera de León A., Sanz H., Elosua R., Guembe M.J., Alzamora M., Vega-Alonso T., Félix-Redondo F.J., Ortiz-Marrón H., Rigo F. (2012). Metabolic syndrome in Spain: Prevalence and coronary risk associated with harmonized definition and WHO proposal. DARIOS study. Rev. Esp. Cardiol..

[4] Bianchi G., Rossi V., Muscari A., Magalotti D., Zoli M., Berzigotti A., Pianoro Study Group (2008). Physical activity is negatively associated with the metabolic syndrome in the elderly. QJM.

[5] Galmes-Panades A.M., Varela-Mato V., Konieczna J., Wärnberg J., Martínez-González M.Á., Salas-Salvadó J. (2019). Isotemporal substitution of inactive time with physical activity and time in bed: Cross-sectional associations with cardiometabolic health in the PREDIMED-Plus study. Int. J. Behav. Nutr. Phys. Act..

[6] Biswas A., Oh P.I., Faulkner G.E., Bajaj R.R., Silver M.A., Mitchell M.S., Alter D.A. (2015). Sedentary time and its association with risk for disease incidence, mortality, and hospitalization in adults a systematic review and meta-analysis. Annals of Internal Medicine. Am. Coll. Phys..

[7] Mankowski R.T., Aubertin-Leheudre M., Beavers D.P., Botoseneanu A., Buford T.W., Church T., Glynn N.W., King A.C., Liu C., Manini T.M. (2015). Sedentary time is associated with the metabolic syndrome in older adults with mobility limitations—The LIFE Study. Exp. Gerontol..

[8] Pan A., Keum N., Okereke O.I., Sun Q., Kivimaki M., Rubin R.R., Hu F.B. (2012). Bidirectional Association Between Depression and Metabolic Syndrome. Diabetes Care.

[9] Estruch R., Martínez-González M.A., Corella D., Salas-Salvadó J., Ruiz-Gutiérrez V., Covas M.I., Fiol M., Gómez-Gracia E., López-Sabater M.C., Vinyoles E. (2006). Effects of a Mediterranean-Style Diet on Cardiovascular Risk Factors a Randomized Trial. Ann. Intern. Med..

[10] Babio N., Toledo E., Estruch R., Ros E., Martínez-González M.A., Castañer O., Bulló M., Corella D., Arós F., Gómez-Gracia E. (2014). Mediterranean diets and metabolic syndrome status in the PREDIMED randomized trial. CMAJ.

[11] DeBoer M.D., Gurka M.J., Woo J.G., Morrison J.A. (2015). Severity of the metabolic syndrome as a predictor of type 2 diabetes between childhood and adulthood: The Princeton Lipid Research Cohort Study. Diabetologia.

[12] Wiley J.F., Carrington M.J. (2016). A metabolic syndrome severity score: A tool to quantify cardio-metabolic risk factors. Prev. Med. (Baltim.).

[13] Martínez-González M.A., Buil-Cosiales P., Corella D., Bulló M., Fitó M., Vioque J., Romaguera D., Martínez J.A., Wärnberg J., López-Miranda J. (2019). Cohort Profile: Design and methods of the PREDIMED-Plus randomized trial. Int. J. Epidemiol..

[14] Alberti K.G.M.M., Eckel R.H., Grundy S.M., Zimmet P.Z., Cleeman J.I., Donato K.A., Fruchart J.C., James W.P.T., Loria C.M., Smith S.S. (2009). International Diabetes Federation Task Force on Epidemiology and Prevention; Hational Heart, Lung, and Blood Institute; American Heart Association; World Heart Federation; International Atherosclerosis Society; International Association for the Study of ObesityHarmonizing the metabolic syndrome: A joint interim statement of the international diabetes federation task force on epidemiology and prevention; National heart, lung, and blood institute; American heart association; World heart federation; International atherosclerosis society; And international association for the study of obesity. Circulation.

[15] Sanz J., Navarro M.E., Vázquez C. (2003). Adaptación española para el Inventario de Depresión de Beck-II (BDI-II). 1. Propiedades psicométricas en estudiantes universitarios. Anál. Modif. Conducta.

[16] Amato M.C., Giordano C. (2014). Visceral adiposity index: An indicator of adipose tissue dysfunction. Int. J. Endocrinol..

[17] Topolski T.D., LoGerfo J., Patrick D.L., Williams B., Walwick J., Patrick M.B. (2006). The Rapid Assessment of Physical Activity (RAPA) among older adults. Prev. Chronic. Dis..

[18] Molina L., Sarmiento M., Peñafiel J., Donaire D., Garcia-Aymerich J., Gomez M., Ble M., Ruiz S., Frances A., Schröder H. (2017). Validation of the regicor short physical activity questionnaire for the adult population. PLoS ONE.

[19] Jones C.J., Rikli R.E., Beam W.C. (1999). A 30-s chair-stand test as a measure of lower body strength in community-residing older adults. Res. Q. Exerc. Sport.

[20] Martínez-González M.A., López-Fontana C., Varo J.J., Sánchez-Villegas A., Martinez J.A. (2005). Validation of the Spanish version of the physical activity questionnaire used in the Nurses’ Health Study and the Health Professionals’ Follow-up Study. Public Health Nutr..

[21] Fernández-Ballart J.D., Piñol J.L., Zazpe I., Corella D., Carrasco P., Toledo E., Perez-Bauer M., Martínez-González M.A., Salas-Salvadó J., Martín-Moreno J.M. (2010). Relative validity of a semi-quantitative food-frequency questionnaire in an elderly Mediterranean population of Spain. Br. J. Nutr..

[22] Moreiras O., Ángeles C., Luisa C., Cuadrado C. (2007). Tabla de Composición de Alimentos.

[23] Galilea-Zabalza I., Buil-Cosiales P., Salas-Salvadó J., Toledo E., Ortega-Azorín C., Díez-Espino J., Vázquez-Ruiz Z., Zomeño M.D., Vioque J., Martínez J.A. (2018). Mediterranean diet and quality of life: Baseline cross-sectional analysis of the PREDIMED-PLUS trial. PLoS ONE.

[24] Shivappa N., Steck S.E., Hurley T.G., Hussey J.R., Hébert J.R. (2014). Designing and developing a literature-derived, population-based dietary inflammatory index. Public Health Nutr..

[25] Guasch-Ferré M., Liu X., Malik V.S., Sun Q., Willett W.C., Manson J.A.E., Rexrode K.M., Li Y., Hu F.B., Bhupathiraju S.N. (2017). Nut Consumption and Risk of Cardiovascular Disease. J. Am. Coll. Cardiol..

[26] Whitman I.R., Agarwal V., Nah G., Dukes J.W., Vittinghoff E., Dewland T.A., Marcus G.M. (2017). Alcohol Abuse and Cardiac Disease. J. Am. Coll. Cardiol..

[27] Kim Y., Je Y. (2018). Meat consumption and risk of metabolic syndrome: Results from the Korean population and a meta-analysis of observational studies. Nutrients.

[28] Takagi H., Hari Y., Nakashima K., Kuno T., Ando T., Alice (All-Literature Investigation of Cardiovascular Evidence) Group (2019). Marriage and mortality after acute coronary syndrome. Eur. J. Prev. Cardiol..

[29] Pucci G., Alcidi R., Tap L., Battista F., Mattace-Raso F., Schillaci G. (2017). Sex- and gender-related prevalence, cardiovascular risk and therapeutic approach in metabolic syndrome: A review of the literature. Pharmacol. Res..

[30] Santilli F., D’Ardes D., Guagnano M.T., Davi G. (2017). Metabolic Syndrome: Sex-Related Cardiovascular Risk and Therapeutic Approach. Curr. Med. Chem..

[31] Xue B., Head J., McMunn A., Heyn P.C. (2019). The Impact of Retirement on Cardiovascular Disease and Its Risk Factors: A Systematic Review of Longitudinal Studies. Gerontologist.

[32] Chen C.C., Li T.C., Chang P.C., Liu C.S., Lin W.Y., Wu M.T., Li C.I., Lin C.C. (2008). Association among cigarette smoking, metabolic syndrome, and its individual components: The metabolic syndrome study in Taiwan. Metabolism.

[33] Syamlal G., Mazurek J.M., Dube S.R. (2014). Gender differences in smoking among U.S. working adults. Am. J. Prev. Med..

[34] Kim I., Song Y.M., Ko H., Sung J., Lee K., Shin J., Shin S. (2018). Educational Disparities in Risk for Metabolic Syndrome. Metab. Syndr. Relat. Disord..

[35] Liu X., Luo X., Liu Y., Sun X., Han C., Zhang L., Wang B., Ren Y., Zhao Y., Zhang D. (2017). Resting heart rate and risk of metabolic syndrome in adults: A dose–response meta-analysis of observational studies. Acta Diabetol..

[36] Womack V.Y., De Chavez P.J., Albrecht S.S., Durant N., Loucks E.B., Puterman E., Redmond N., Siddique J., Williams D.R., Carnethon M.R. (2016). A Longitudinal Relationship between Depressive Symptoms and Development of Metabolic Syndrome: The Coronary Artery Risk Development in Young Adults Study. Psychosom. Med..

[37] Kazaz I., Angin E., Kabaran S., Iyigün G., Kirmizigil B., Malkoç M. (2018). Evaluation of the physical activity level, nutrition quality, and depression in patients with metabolic syndrome. Medicine (Baltim.).

[38] Grøntved A., Hu F.B. (2011). Television viewing and risk of type 2 diabetes, cardiovascular disease, and all-cause mortality: A meta-analysis. JAMA J. Am. Med. Assoc..

[39] Bankoski A., Harris T.B., McClain J.J., Brychta R.J., Caserotti P., Chen K.Y., Berrigan D., Troiano R.P., Koster A. (2011). Sedentary activity associated with metabolic syndrome independent of physical activity. Diabetes Care.

[40] Strasser B. (2013). Physical activity in obesity and metabolic syndrome. Ann. N. Y. Acad. Sci..

[41] Serrano-Sánchez J.A., Fernández-Rodríguez M.J., Sanchis-Moysi J., del Rodríguez-Pérez M.C., Marcelino-Rodríguez I., de León A.C. (2019). Domain and intensity of physical activity are associated with metabolic syndrome: A population-based study. PLoS ONE.

[42] Ekelund U., Steene-Johannessen J., Brown W.J., Fagerland M.W., Owen N., Powell K.E., Bauman A., Lee I.M., Lancet Physical Activity Series 2 Executive Committe, Lancet Sedentary Behaviour Working Group (2016). Does physical activity attenuate, or even eliminate, the detrimental association of sitting time with mortality? A harmonised meta-analysis of data from more than 1 million men and women. Lancet.

[43] Pedrero-Chamizo R., Gómez-Cabello A., Delgado S., Rodríguez-Llarena S., Rodríguez-Marroyo J.A., Cabanillas E., Meléndez A., Vicente-Rodríguez G., Aznar S., Villa G. (2012). Physical fitness levels among independent non-institutionalized Spanish elderly: The elderly EXERNET multi-center study. Arch. Gerontol. Geriatr..

[44] Widmer R.J., Flammer A.J., Lerman L.O., Lerman A. (2015). The Mediterranean diet, its components, and cardiovascular disease. Am. J. Med..

[45] Namazi N., Larijani B., Azadbakht L. (2018). Dietary Inflammatory Index and its Association with the Risk of Cardiovascular Diseases, Metabolic Syndrome, and Mortality: A Systematic Review and Meta-Analysis. Horm. Metab. Res..

[46] Grandl G., Wolfrum C. (2018). Hemostasis, endothelial stress, inflammation, and the metabolic syndrome. Semin. Immunopathol..

[47] Abete I., Romaguera D., Vieira A.R., De Lopez Munain A., Norat T. (2014). Association between total, processed, red and white meat consumption and all-cause, CVD and IHD mortality: A meta-analysis of cohort studies. Br. J. Nutr..

[48] Mirmiran P., Ziadlou M., Karimi S., Hosseini-Esfahani F., Azizi F. (2019). The association of dietary patterns and adherence to WHO healthy diet with metabolic syndrome in children and adolescents: Tehran lipid and glucose study. BMC Public Health.

[49] Hooper L., Martin N., Abdelhamid A., Davey Smith G. (2015). Reduction in saturated fat intake for cardiovascular disease. Cochrane Database Syst. Rev..

[50] Oteng A.-B., Kersten S. (2019). Mechanisms of Action of trans Fatty Acids. Adv. Nutr..

[51] Lorente-Cebrián S., Costa A.G.V., Navas-Carretero S., Zabala M., Martínez J.A., Moreno-Aliaga M.J. (2013). Role of omega-3 fatty acids in obesity, metabolic syndrome, and cardiovascular diseases: A review of the evidence. J. Physiol. Biochem..

[52] Hyde P.N., Sapper T.N., Crabtree C.D., LaFountain R.A., Bowling M.L., Buga A., Fell B., McSwiney F.T., Dickerson R.M., Miller V.J. (2019). Dietary carbohydrate restriction improves metabolic syndrome independent of weight loss. JCI Insight.

[53] Hosseinpour-Niazi S., Hosseini S., Mirmiran P., Azizi F. (2017). Prospective study of nut consumption and incidence of metabolic syndrome: Tehran Lipid and glucose study. Nutrients.

[54] Soriguer F., García-Fuentes E., Gutierrez-Repiso C., Rojo-Martínez G., Velasco I., Goday A., Bosch-Comas A., Bordiú E., Calle A., Carmena R. (2012). Iodine intake in the adult population. Di@bet.es study. Clin. Nutr..

[55] Sparks E., Farrand C., Santos J.A., McKenzie B., Trieu K., Reimers J., Davidson C., Johnson C., Webster J. (2018). Sodium Levels of Processed Meat in Australia: Supermarket Survey Data from 2010 to 2017. Nutrients.

[56] Merino J., Guasch-Ferré M., Martínez-González M.A., Corella D., Estruch R., Fitó M., Ros E., Arós F., Bulló M., Gómez-Gracia E. (2015). Is complying with the recommendations of sodium intake beneficial for health in individuals at high cardiovascular risk? Findings from the PREDIMED study. Am. J. Clin. Nutr..

[57] Valerio G., Mozzillo E., Zito E., De Nitto E., Maltoni G., Marigliano M., Zucchini S., Maffeis C., Franzese A. (2019). Alcohol consumption or cigarette smoking and cardiovascular disease risk in youth with type 1 diabetes. Acta Diabetol..

